# Naval Lunatics at Haslar

**Published:** 1844-01

**Authors:** 


### The Naval Lunatics at Haslar.

In no situation has the humane system of treating lunatics been carried out with greater vigour than in the Royal Naval Hospital at Haslar, and nowhere with more satisfactory and gratifying results. So soon as the happy consequences of Dr. Conolly's new system at Hanwell came to the knowledge of the Director-General of the Medical Department of the Navy, Sir William Burnett, he determined on introducing it into the government asylum at Haslar; and he had the good fortune to find in one of the officers of his own service, Dr. Anderson, a man not merely fully convinced of its immense superiority, but practically conversant with its details. For some time previously to his appointment, Dr. Anderson had been the resident physician of the well-known private institution at Denham Park, near Uxbridge, where the non-restraint system was and is carried to its full extent. At the period of Dr. Anderson's appointment, the naval lunatics, both men and officers, were treated pretty much in the old plan, although Sir William Burnett had made strenuous efforts for the introduction of the more rational system. Dr. Anderson's arrival at Haslar was the epoch of a complete reform; and as his views coincided in every respect with those of Sir William Burnett, and as that officer had the entire confidence of the Lords of the Admiralty (who entered into the question with a degree of zeal and liberality highly honorable to themselves), every projected improvement was carried into effect with true nautical energy. The change for the better was the more remarkable for its rapidity. Those who had known the asylum under the old rule could scarcely credit their senses when witnessing, after a few months, the new discipline of the place and the new habits and dispositions of the inmates. Chains, straps, corsets, imprisonment—all vanished at the will of the Superintendent; and the false fears of the attendants, and much of the gloom and misery of the patients, soon followed. This altered condition of things has now existed about two years, and not a single accident has occurred to checker the satisfaction of those who brought it about.

Among the happy changes introduced by Dr. Anderson was one suggested by Sir William Burnett, which does great credit to his humanity, and has been a source of immense gratification to the patients. The airing-court attached to the asylum being at the back of the hospital, which stands on flat ground, was necessarily deprived of all prospect of the surrounding country, and even of the neighbouring sea. To remedy this defect it was determined to erect a lofty mound in the centre of the airing ground; and this was no sooner suggested than it was carried into effect by the lunatics themselves, under the direction of their benevolent superintendent. This mound is of large dimensions, solidly and beautifully constructed, and furnished with a gravel-walk leading by a gentle slope to the summit. It is sufficiently lofty to command a very extensive and beautiful view of the Isle of Wight as far as Cowes and St. Helen's, the towns of Portsmouth and Portsea, great part of Portsmouth harbour, all Spithead, and the neighbouring sea, with the ever-busy and ever-shifting panorama of masts and sails and flags that crowd that nautical thoroughfare. It is impossible to contemplate, without emotion, the happy influence that such a change as this must have had on the feelings of the sailors. The famous shout of Xenophon's soldiers, "The Sea! the Sea!" seems again realized to the imagination when we think of the feelings that may have stirred the withered souls of those solitary men, when, after years of imprisonment within gloomy walls, they were again, as it were, restored to their old element. How much they prize the privilege thus accorded by the purest and most refined humanity, is proved by the continued eagerness displayed by them in climbing this "sacred mount." A similar elevation is now being built in the grounds of the officers' department.

A still bolder step in the progress of the rational and humane treatment of these poor fellows, and, we doubt not, in the cure of their bruised and broken minds,—an improvement which strikes us almost as much by its happy boldness as by its genuine benevolence,—has been more recently introduced. A boat has been granted to them by the Admiralty; and in this they may now be seen pulling and steering their fearless and noble-hearted friend and master, Dr. Anderson, not only through Portsmouth harbour, but actually out to sea, calmly enjoying the cooling breeze, or busy in their long-forgotten pastime of fishing.

But the account of this affecting experiment, and a few more interesting particulars of the proceedings of the Naval Asylum, we are enabled, through the kindness of Sir William Burnett, to give in Dr. Anderson's own words; and we cannot present them to our readers without offering to that gentleman the tribute of our respect and gratitude for his enlightened and noble exertions.

*Extract from Dr. Anderson's Report, Midsummer Quarter*, 1843.—"The calm and orderly conduct which now prevails throughout the asylum, together with the cleanly, and I may add industrious, habits of a large proportion of the patients, render the duties of the attendants and nurses comparatively easy. For the accomplishment of these desirable ends I have endeavoured to carry out the excellent precepts laid down by Dr. Conolly of Hanwell; and in order to point out clearly the principles by which the medical officers themselves are guided, and that they constantly inculcate on the attendants and nurses, I cannot do better than quote the following paragraph from one of that talented physician's Reports. 'To endeavour to gain and preserve the confidence of each patient; to create or maintain a character of kindness and tranquillity throughout the asylum; to forbid the exercise of violence, threats, or deception; to be careful of their diet and clothing; to occupy and amuse them; to secure their cheerfulness or content by day and comfortable rest at night; to consider all their weaknesses and infirmities; and to pay a general regard to whatever may act favorably on the mind and body.' These have been and continue to be the principles on which our moral management is based, and the fruits of this mild and rational treatment are now clearly developed in the altered condition of our inmates, whose conduct for many months past has very generally been characterized by a constant and orderly demeanour, which I am persuaded no coercive measures could ever have produced. The means of recreation and exercise which have wisely been extended to the patients in allowing them to walk into the surrounding country, continue to be a source of great delight to many; and nothing is felt as a more severe punishment than this salutary freedom being withheld from any of those who are usually in the habit of joining the party in these country excursions. The cheerful aspect of the new airing-grounds with the mound in the centre, has been very much increased by the late alterations; and being now thrown open in fine weather, have become the daily resort of a large proportion of the patients. The inducement thus offered to the indolent and melancholic to take a view of Spithead, Portsmouth harbour, Isle of Wight, and the surrounding country, is found not only to form the means of amusement and exercise, but, it may almost be said, some alleviation of their malady.

"The religious services continue to be regularly performed in the asylum morning and evening; and it is rather surprising to find between sixty and seventy insane persons assembled together for the purpose of divine worship without the occurrence of any disturbance, except on very rare occasions. Upwards of twenty attend the service in the chapel every Sunday; and their conduct during divine worship has been uniformly marked by the same decorum as the sane part of the congregation.

"In consequence of directions from the Inspector-General, the dress for the seamen and marines was changed at Easter, the whole of the lunatic patients being now supplied with blue clothing instead of the brown formerly worn. This change to their favourite colour has given the seamen great satisfaction; and no doubt has in some of the number brought back to their recollection early and pleasing associations.

"The many alterations, sanctioned and promoted by the Inspector-General, which have taken place generally throughout the asylum during the last twelve months, have contributed in no small degree to the improved condition of the patients; and when the works now in progress are completed, the whole face of the establishment will be changed from its former gloomy and prison-like appearance to a cheerful place of residence, which will neither be calculated to create alarm in the timid and suspicious, nor excite the rage of the more furious madman."

*Extract from Dr. Anderson's Report, Michaelmas Quarter*, 1843.—"The Lords of the Admiralty have very recently, on the recommendation of Sir William Burnett, furnished a boat for the use of the lunatic patients; and since it has been received from the dockyard they have had many rowing and several sailing excursions, both in the harbour and out to Spithead. Upwards of twenty of the patients have, at different times, joined in this amusement; and on every occasion they have behaved in the most quiet and orderly manner, and can now, after the practice of some weeks, manage the boat either under sail or in rowing with perfect ease and dexterity. I shall not attempt to describe the enjoyment which many have felt by being again permitted to embark on their favorite element, nor the pleasure with which they look forward to these water-excursions. Some of those who have proved to be our best boatmen have been confined within the narrow limits of the asylum airing-grounds for upwards of twenty years; but, notwithstanding this protracted period of seclusion, their new occupation appears to have roused mental energies which to the common observer were nearly extinct, and to have brought back to their enfeebled minds old and pleasing associations which have been productive of the most beneficial results. I have no hesitation in stating that of all the remedial agents with which we have during the last eighteen months been so liberally supplied, the use of the boat for many of our patients is, beyond all comparison, the most valuable of any; and my anticipations, sanguine as they were as to the benefits likely to result from that kind of exercise and recreation on the mind of the lunatic sailor, have been most fully and completely realized.

"The Inspector-General has also directed a supply of fishing lines and hooks to be furnished, and the patients have frequently, during the last month, been successfully occupied in fishing, which is also a source of great enjoyment and interest to many. Mr. * * * affords, among many others, a striking example of the salutary influence resulting from the new occupation. This officer has been in the asylum for eight years, and during the last six has very rarely spoken to any one, and appeared to take little or no notice of surrounding objects. The various means that were had recourse to with the view of rousing him from his apathetic state proved fruitless. He, in common with others, was furnished with a fishing line; and on his first trip to the buoy of the Boyne, which is our principal fishing ground, caught nine whiting-poult, and enjoyed the sport as intensely as any one in the boat, baiting his hooks himself, and making observations on his success. On our way home he took all the fish that were caught out of the basket and *counted them aloud*, much to the astonishment of the steward and myself, as well as to others who had scarcely heard the sound of his voice for several years. It is a pleasing part of my duty to be thus enabled to report the complete success of a measure which, in the estimation of many, was fraught with so much personal danger to the lunatic, by affording him an easy opportunity of carrying any suicidal propensity into effect; but the truth is, that, in well-regulated establishments for the reception of the insane, the tendency to self-destruction is nearly, if not altogether, as rare as amongst those who are considered the sane part of the community.

"It is scarcely necessary to state that no bodily restraint has been had recourse to; but, as was explained to the Inspector-General during his recent official visit, it has frequently happened that for many days in succession not a single individual has been placed in temporary seclusion, thus clearly demonstrating the many advantages resulting from a mild and conciliatory system of management as compared with harsh and coercive measures.

"In conclusion, I may be permitted to state the extreme gratification I have felt in witnessing the progressive but steady improvement in the general demeanour, the cleanly habits, and the contented appearance of many whose condition, twelve months ago, scarcely held out, under any treatment, the prospect of such cheering results."

We think that no one after reading the foregoing account, will hesitate to place the name of ANDERSON high on the list of those noblest philanthropists who are proud to own Dr. Conolly as their chief. Through his exertions and those of Sir William Burnett, we are justified in saying of HASLAR what Professor Marx so finely said of HANWELL: and we think the Professor's words, which we once proposed as a motto for Hanwell, may with full propriety be now inscribed on the walls of Haslar:—"HIER WIRD DURCH DIE THAT BEWIESEN WAS DER MENSCH ÜBER DEN MENSCHEN DURCH DAS MENSCHLICHE VERMAG."

